# Clinical Diagnosis: The Bacteriological, Chemical and Microscopical Evidence of Disease

**Published:** 1898-01

**Authors:** 


CLINICAL DIAGNOSIS: The Bacteriological, Chemical and Microscopical Evidence of Disease. By DR. RUDOLF v. JAKSCH, Professor of Special Pathology and Therapeutics and Director of the Medical Clinic in the German University of Prague. Translated from the fourth German edition and enlarged by James Cagney, M. A., M. D., Member of the Royal College of Physicians of London; Physician to the Hospital for Epilepsy and Paralysis, etc. Third edition. Octavo, pp. xxv.—523. With numerous illustrations (partly in colors). London: Charles Griffin & Company, Limited, Exeter street, Strand. 1897

The appearance of the third English translation of the fourth edition of this classical work on Clinical diagnosis shows the popularity and esteem in which it is held by the continental and English physicians. American physicians are beginning to appreciate the value and efficiency of this work and well they may, for it stands for all that is exact and authoritative in the most important branch of the medical sciences. The thoroughness of the work may be judged somewhat by the references to literature. Thus, in the chapter on the urine, 770 references are given, while on the blood 383 references are noted.

Chapter I. treats of the blood, the author discussing the color, reaction, specific gravity, changes in the formed elements of the blood, parasites of the blood and the chemical changes. The author describes carefully all the various instruments for estimating the specific gravity, amount of hemoglobin and the number of red and white corpuscles. Chapter II. is devoted to the buccal secretions, the coatings on the tongue and teeth, and the condition of the saliva in certain diseases. Chapter III. treats very briefly of the nasal secretion. Chapter IV. takes up the sputum, the microscopical and bacteriological examinations and the character and constituents of the sputum in the principal diseases of the lungs and bronchi. Chapter V. discusses at some length the gastric juice and vomit. Chapter VI. treats of the feces. Chapter VII., the examination of the urine. Chapter VIII., the investigation of exudations, transudations and cystic fluids. Chapter IX., the secretions of the genital organs, and Chapter X. of the methods of bacteriological research. A very complete bibliography of sixty-two pages follows and then a good index. There are 157 illustrations, nicely executed, many in colors. For those who wish to consult all that has been published of merit on the subject of clinical diagnosis, no better work can be recommended than v. Jaksch's.

The work is well printed on good paper, but deserves a better binding. W. C. K.

